# Stimulation of Inositol 1,4,5-Trisphosphate (IP_3_) Receptor Subtypes by Adenophostin A and Its Analogues

**DOI:** 10.1371/journal.pone.0058027

**Published:** 2013-02-28

**Authors:** Huma Saleem, Stephen C. Tovey, Andrew M. Riley, Barry V. L. Potter, Colin W. Taylor

**Affiliations:** 1 Department of Pharmacology, Cambridge, United Kingdom; 2 Wolfson Laboratory of Medicinal Chemistry, Department of Pharmacy and Pharmacology, University of Bath, Bath, United Kingdom; Indiana University School of Medicine, United States of America

## Abstract

Inositol 1,4,5-trisphosphate receptors (IP_3_R) are intracellular Ca^2+^ channels. Most animal cells express mixtures of the three IP_3_R subtypes encoded by vertebrate genomes. Adenophostin A (AdA) is the most potent naturally occurring agonist of IP_3_R and it shares with IP_3_ the essential features of all IP_3_R agonists, namely structures equivalent to the 4,5-bisphosphate and 6-hydroxyl of IP_3_. The two essential phosphate groups contribute to closure of the clam-like IP_3_-binding core (IBC), and thereby IP_3_R activation, by binding to each of its sides (the α- and β-domains). Regulation of the three subtypes of IP_3_R by AdA and its analogues has not been examined in cells expressing defined homogenous populations of IP_3_R. We measured Ca^2+^ release evoked by synthetic adenophostin A (AdA) and its analogues in permeabilized DT40 cells devoid of native IP_3_R and stably expressing single subtypes of mammalian IP_3_R. The determinants of high-affinity binding of AdA and its analogues were indistinguishable for each IP_3_R subtype. The results are consistent with a cation-π interaction between the adenine of AdA and a conserved arginine within the IBC α-domain contributing to closure of the IBC. The two complementary contacts between AdA and the α-domain (cation-π interaction and 3″-phosphate) allow activation of IP_3_R by an analogue of AdA (3″-dephospho-AdA) that lacks a phosphate group equivalent to the essential 5-phosphate of IP_3_. These data provide the first structure-activity analyses of key AdA analogues using homogenous populations of all mammalian IP_3_R subtypes. They demonstrate that differences in the Ca^2+^ signals evoked by AdA analogues are unlikely to be due to selective regulation of IP_3_R subtypes.

## Introduction

**Figure 1:**
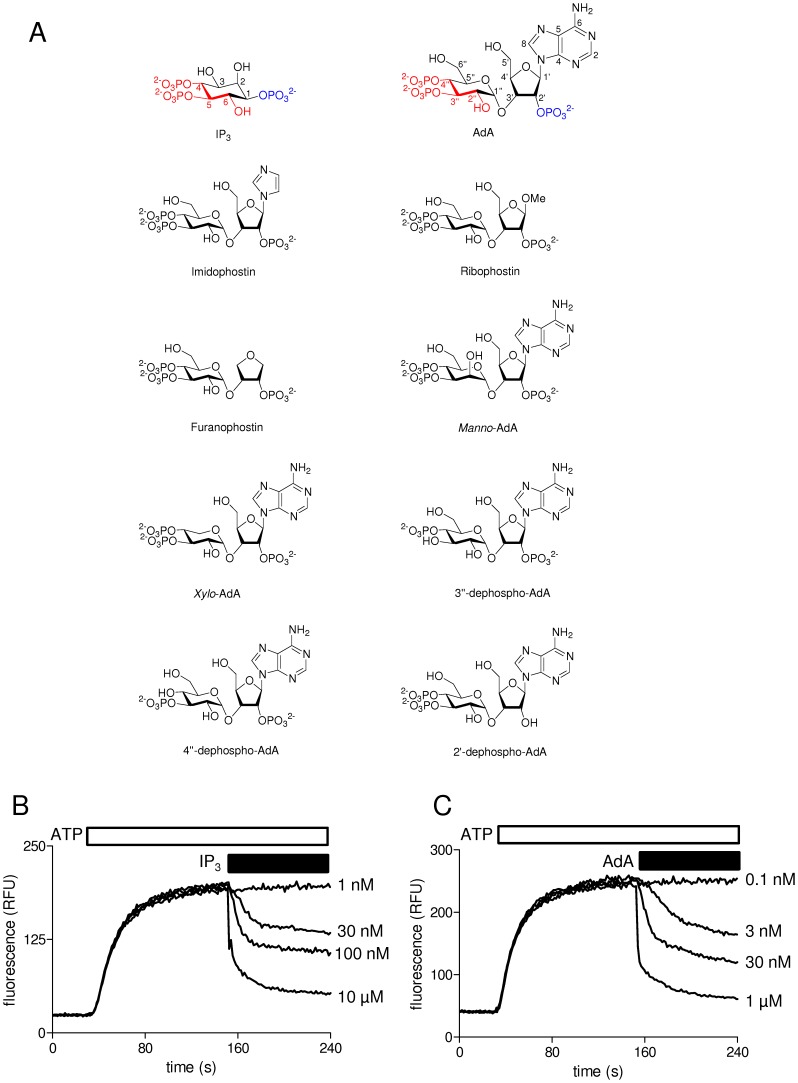
Structures of the analogues of AdA used. (A) Key moieties within IP_3_ and AdA are highlighted in matching colours to indicate their proposed structural equivalence. (B and C). The Ca^2+^ contents of the intracellular stores of populations of permeabilized DT40-IP_3_R1 cells are shown after addition of ATP to allow active Ca^2+^ uptake, and then addition of the indicated concentrations of IP_3_ or AdA with thapsigargin (1 µM) to inhibit further Ca^2+^ uptake. The traces, which are typical of those from all subsequent analyses, show the average response from 2 wells on a single plate. The results demonstrate that both IP_3_ and AdA evoke quantal Ca^2+^ release.

Inositol 1,4,5-trisphosphate receptors (IP_3_R) are intracellular Ca^2+^ channels that are expressed in almost all animal cells. They allow release of Ca^2+^ from intracellular stores in response to the many stimuli that activate phospholipase C [1], [2]. The genomes of vertebrates encode three closely related IP_3_R subtypes (IP_3_R1-3), and most cells from vertebrates express functional IP_3_R that are homo- or hetero-tetrameric assemblies of these IP_3_R subtypes and their splice variants [3]. The physiological significance of this IP_3_R diversity is poorly understood, and nor are there ligands that usefully discriminate between IP_3_R subtypes. It is, however, clear that activation of IP_3_R is initiated by binding of IP_3_ to the conserved IP_3_-binding core (IBC, residues 224-604 of IP_3_R1) of each IP_3_R subunit [4]. Mixed populations of IP_3_R in native cells make it difficult to define unambiguously the functional properties of each IP_3_R subtype. Stable heterologous expression of mammalian IP_3_R in the only vertebrate cell line engineered to lack all endogenous IP_3_R (DT40 KO cells) [5] provides an effective means of addressing this difficulty [6]. We previously used DT40 cells expressing homogeneous populations of each mammalian IP_3_R subtype to define structure-activity relationships for key endogenous and synthetic inositol phosphates [7]. Here, we extend the approach to examine the interactions of each IP_3_R subtype with adenophostin A (**1**, AdA) and its most important analogues [8] (Figure 1A).

AdA, originally isolated from *Penicillium brevicompactum* [9], [10] and later synthesized [11], is a potent agonist of IP_3_R. It is also resistant to degradation by the enzymes that degrade IP_3_ via phosphorylation or dephosphorylation [10]. Although AdA is based on a glucose ring, rather than the inositol ring of IP_3_, its structure retains the key functional groups of IP_3_ that are known to be essential for IP_3_ activity at IP_3_R [12] (Figure 1A). Considerable evidence supports the original suggestion [10] that the essential 4,5-bisphosphate and 6-hydroxyl of IP_3_ are effectively mimicked by the 4″,3″-bisphosphate and 2″-hydroxyl of AdA (red highlights in Figure 1A). The interactions that allow AdA to bind to IP_3_R with about 10-fold greater affinity than IP_3_ have been more difficult to resolve. One view was that the 2′-phosphate of AdA is equivalent to the 1-phosphate of IP_3_ and, like the latter [13] (blue in Figure 1A), contributes to high-affinity binding to the IBC. The suggestion was that the 2′-phosphate of AdA forms a stronger interaction with the IBC than does the 1-phosphate of IP_3_. Our recent analyses have challenged this idea and instead suggest that a cation-π interaction between the adenine ring of AdA and a guanidinium side chain of an arginine residue within the α-domain of the IBC (R504 in IP_3_R1) may be a more important determinant of the increased affinity of AdA for IP_3_R [12].

The high-affinity and metabolic stability of AdA have generated considerable interest in both the synthesis of AdA analogues and their application to analyses of IP_3_R activation and associated changes in cytosolic Ca^2+^ signalling [12]. There has, however, been no systematic analysis of the activities of AdA or its analogues with defined populations of homogenous IP_3_R subtypes. The need for such analyses is particularly important in attempting to explain results in which Ca^2+^ signals evoked by IP_3_ differ from those evoked by AdA [14], [15], [16], [17], [18], [19], [20], [21], or where different analogues of AdA evoke different cellular responses [reviewed in 12,22]. Here we use DT40 cells in which all endogenous IP_3_R have been genetically inactivated [5] to stably express homogenous populations of mammalian IP_3_R subtypes and thereby define structure-activity relationships for AdA and its key analogues for each IP_3_R subtype.

## Materials and Methods

### Materials

Sources of most reagents were provided in a previous publication [7]. The structures of the ligands used and their abbreviations are shown in Figure 1A. IP_3_ was from Alexis Biochemicals (Nottingham, UK). AdA [23], imidophostin [24], ribophostin [25], furanophostin [26], *manno*-AdA and *xylo*-AdA [27], 3″-dephospho AdA and 4″-dephospho AdA [28], and 2′-dephospho AdA were synthesized, purified and characterized as previously described.

### Measurement Ca^2+^ Release by IP_3_ Receptors

From quantitative analyses of western blots using antisera that selectively recognise each IP_3_R subtype or react equally with all three subtypes, we established that in the DT40 cells used, levels of IP_3_R expression (relative to IP_3_R3) were IP_3_R1 (71±8%, n = 3), IP_3_R2 (48±5%) and IP_3_R3 (100%) [7]. It is impracticable to achieve identical levels of IP_3_R expression for each cell line, and differences (albeit modest in our cell lines) may affect both the size of the IP_3_-sensitive Ca^2+^ pool and its sensitivity to IP_3_ [29]. The different levels of IP_3_R expression do not compromise the analyses reported here, which are entirely concerned with relative potencies of AdA analogues for each IP_3_R subtype (see below).

A comprehensive description of the methods used to measure free [Ca^2+^] within the endoplasmic reticulum of permeabilized DT40 cells was provided in preceding publications [7], [30]. Briefly, the endoplasmic reticulum of DT40 cells stably expressing each of the three mammalian IP_3_R subtypes was loaded with a low-affinity Ca^2+^ indicator (Mag fluo-4) [30]. After permeabilization of the plasma membrane with saponin (10 µg/mL, ∼4 min, 37°C), the permeabilized cells in cytosol-like medium (CLM) were distributed into 96-well plates at 20°C. Addition of MgATP (1.5 mM) then allowed active Ca^2+^ accumulation, which was monitored at intervals of ∼1 s using a FlexStation 3 fluorescence plate-reader (MDS Analytical Devices). CLM had the following composition: 140 mM KCl, 20 mM NaCl, 1 mM EGTA, 20 mM Pipes, pH 7, free [Ca^2+^] ∼220 nM (after addition of MgATP), and carbonyl cyanide 4-trifluoromethoxy-phenyl hydrazone (FCCP, 10 µM) to inhibit mitochondrial Ca^2+^ uptake. After 150 s, when the stores had loaded to steady-state with Ca^2+^, IP_3_, AdA or its analogues was added with thapsigargin (1 µM) to prevent further Ca^2+^ uptake, and after a further 30 s, the response was recorded. Agonist-evoked Ca^2+^ release was expressed as a fraction of that released by ionomycin (1 µM) [30]. All experiments were performed at 20°C.

### Statistical Analysis

Concentration-effect relationships were fitted to Hill equations using GraphPad Prism (version 5.0) from which Hill coefficients (h), the fraction of the intracellular Ca^2+^ stores released by maximally effective concentrations of agonist, and pEC_50_ values (-log EC_50_) were calculated. For convenience some results are presented as EC_50_ values, but all statistical comparisons use pEC_50_ values. Within each experiment, the pEC_50_ for AdA was determined to allow paired comparisons with values obtained for each AdA analogue. These are reported as ΔpEC_50_, where:
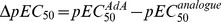



We note that Table 1 reports pooled results from experiments collected over a considerable period, whereas ΔpEC_50_ values, like those shown in Table 2, compare only paired values. The latter provide the most robust means of comparing agonist potencies. Results are expressed as means ± SEM from n independent experiments, with each experiment performed in triplicate.

**Table 1:** Effects of AdA analogues on Ca^2+^ release by subtypes of IP_3_ receptor.

	IP_3_R1					IP_3_R2					IP_3_R3				
	EC_50_	pEC_50_	h	Ca^2+^release	n	EC_50_	pEC_50_	h	Ca^2+^release	n	EC_50_	pEC_50_	h	Ca^2+^release	n
(1,4,5)IP_3_	87	7.06±0.05	0.99±0.05	75±1	31	145	6.84±0.06	1.29±0.09	61±2	34	417	6.38±0.05	1.26±0.07	64±2	30
AdA	8.3	8.08±0.09	1.17±0.09	72±3	10	18.2	7.74±0.06	1.79±0.21	56±2	13	33	7.48±0.09	1.13±0.07	61±2	14
Imidophostin	37	7.43±0.28	1.17±0.21	78±5	3	68	7.17±0.14	1.84±0.50	59±3	3	166	6.78±0.16	1.73±0.39	67±7	3
Ribophostin	40	7.40±0.29	1.34±0.16	77±4	3	102	6.99±0.11	1.60±0.50	61±2	3	295	6.53±0.21	1.42±0.08	68±4	3
Furanophostin	51	7.29±0.25	0.90±0.10	79±6	3	76	7.12±0.01	1.73±0.20	60±3	3	457	6.34±0.18	1.27±0.21	71±3	3
*Manno*-AdA	34	7.47±0.19	1.33±0.30	75±7	3	69	7.16±0.07	1.33±0.22	57±3	3	245	6.61±0.23	1.23±0.15	69±4	3
*Xylo*-AdA	5.9	8.23±0.17	1.27±0.27	73±7	3	7.9	8.10±0.10	1.52±0.40	52±6	3	29	7.54±0.12	1.58±0.29	64±9	3
2′-dephospho-AdA	275	6.56±0.13	1.31±0.15	66±7	3	575	6.24±0.10	0.85±0.07	63±2	3	692	6.16±0.03	1.5±0.22	55±7	4
3″-dephospho-AdA^c^	ND	ND	ND	15±6^b^	7	ND	ND	ND	6±2^b^	6	ND	ND	ND	7±7^b^	5
4″-dephospho-AdA	Inactive^a^	Inactive^a^	Inactive^a^	ND	6	Inactive^a^	Inactive^a^	Inactive^a^	ND	6	Inactive^a^	Inactive^a^	Inactive^a^	ND	5

The EC_50_ (nM), pEC_50_ (/M), Hill coefficient (h) and fraction (%) of the intracellular Ca^2+^ stores released by a maximally effective concentration of each analogue are shown for each IP_3_R subtype. All results (except EC_50_) are shown as means ± SEM from n independent experiments.

a Inactive at 300 µM.

b Ca^2+^ release evoked by 300 µM 3″-dephospho AdA.

c Refer to Table 2 for relative potencies of 3″-dephospho AdA. ND, not determined.

**Table 2:** Relative potencies of AdA analogues at different IP_3_ receptor subtypes.

	IP_3_R1	IP_3_R2	IP_3_R3
IP_3_	1.02±0.02	0.9±0.30	1.1±0.30
Imidophostin	0.78±0.15	0.78±0.08	0.81±0.04
Ribophostin	0.82±0.18	0.96±0.20	1.06±0.07
Furanophostin	0.92±0.13	0.83±0.14	1.25±0.05
*Manno*-AdA	0.74±0.08	0.79±0.18	0.98±0.08
*Xylo*-AdA	−0.01±0.07	−0.3±0.27	0.05±0.08
2′-dephospho-AdA	1.24±0.33	1.60±0.18	1.68±0.16
3″-dephospho-AdA^a^	4.03±0.09	4.47±0.30	4.13±0.14

From paired comparisons with AdA, the potency (ΔpEC_50_) of the analogues relative to AdA is shown for each IP_3_R subtype. Results are means ± SEM, with n provided in Table 1. ND, not determined. ^a^Because the very low affinity of 3″-dephospho AdA for IP_3_R made it impracticable to stimulate cells with a maximally effective concentration, ‘ΔpEC_50_’ for 3″-dephospho AdA was estimated by comparing concentrations of it and AdA that evoked the same sub-maximal Ca^2+^ release.

Statistical comparisons used Student’s t-test or ANOVA followed by Bonferroni’s *post hoc* test, as appropriate, with *P*<0.05 considered significant. Because not all comparisons of the relative potencies of AdA and IP_3_ were paired, the SEM of this ΔpEC_50_ value was calculated from:
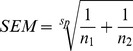
where, s_p_ is the estimate of the population variance:



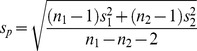
where, s_1_ and s_2_ are the sample standard deviations, and n_1_ and n_2_ are the sample sizes [31].

## Results

### Quantal Ca^2+^ Release Evoked by AdA and IP_3_

The kinetics of IP_3_-evoked Ca^2+^ release from intracellular stores are unexpectedly complex. It is widely observed that under conditions where Ca^2+^ uptake into the endoplasmic reticulum (ER) is inhibited, submaximally effective concentrations of IP_3_ rapidly release only a fraction of the IP_3_-sensitive Ca^2+^ stores [32]. Thereafter, there is either no, or a massively reduced, effect of IP_3_ on the rate of Ca^2+^ release. The mechanisms underlying this pattern of response, known as quantal Ca^2+^ release [33], remain unclear. It may require desensitization of IP_3_R as the Ca^2+^ content of the ER declines [34] or heterogeneity among IP_3_-senstive Ca^2+^ stores [35]. The results shown in Figures 1B and C confirm that the Ca^2+^ release evoked by submaximal concentrations of either IP_3_ or AdA from permeabilized DT40-IP_3_R1 cells is quantal. These observations provide the justification for all subsequent experiments in which the concentration-dependent effects of IP_3_ or AdA were measured 30 s after their addition (see Methods).

### AdA is a Potent Agonist of All Three IP_3_ Receptor Subtypes

**Figure 2:**
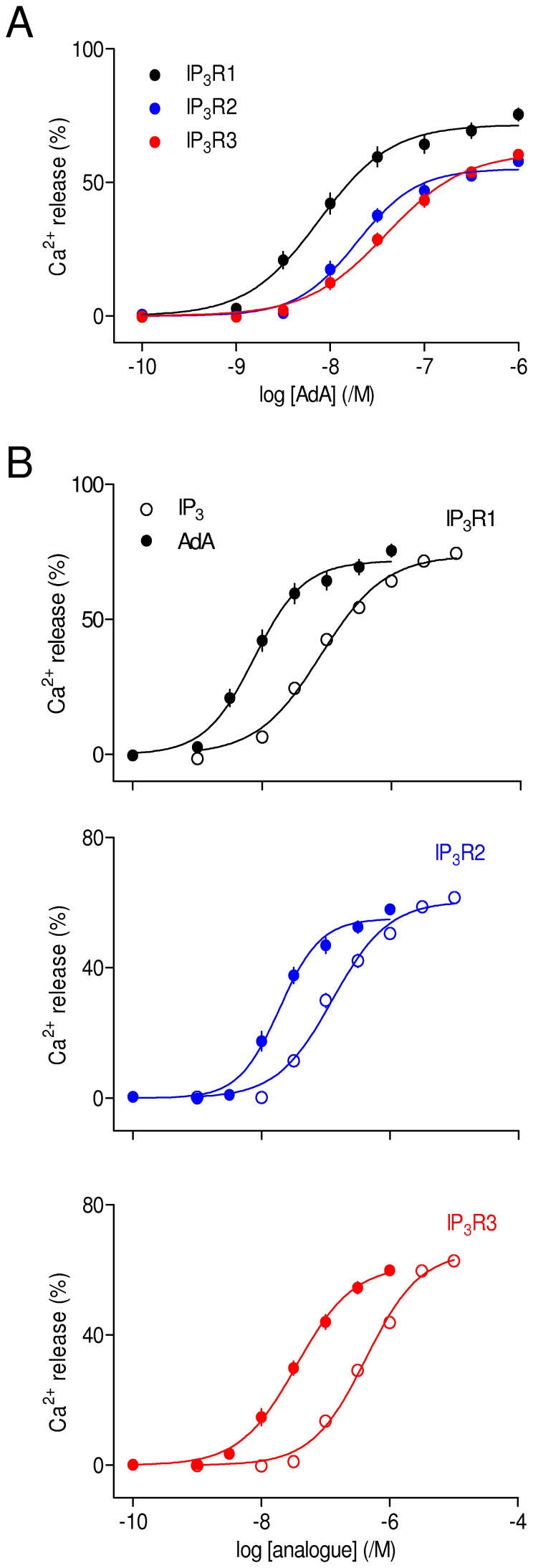
AdA is a potent agonist of all three IP_3_ receptor subtypes. (A) Concentration-dependent effects of AdA on Ca^2+^ release from the intracellular stores of cells expressing IP_3_R1, IP_3_R2 or IP_3_R3. All results are expressed as percentages of the Ca^2+^ release evoked by ionomycin. The same colour codes are used in all subsequent figures. (B) Comparison, for each IP_3_R subtype, of the Ca^2+^ release evoked by IP_3_ and AdA. Results are means ± SEM from the number of independent experiments given in Table 1. Here, and in many subsequent figures, some error bars are smaller than the symbols.

The results shown in Figure 2 and Tables 1 and 2 demonstrate that AdA is ∼10-times more potent than IP_3_ at each IP_3_R subtype, and for each subtype, maximally effective concentrations of IP_3_ and AdA release the same fraction of the intracellular Ca^2+^ stores. This is consistent with many analyses of IP_3_ and AdA in a variety of cell types using both functional and binding assays, in which AdA behaves as a full agonist with ∼10-fold greater affinity than IP_3_ [reviewed in 8]. Our results do, however, provide the first direct demonstration that AdA interacts similarly with all three IP_3_R subtypes. Subsequent experiments examine the interactions between key analogues of IP_3_ and AdA with each IP_3_R subtype.

### Trimming the Adenosine Moiety of AdA Reduces its Potency at All IP_3_ Receptor Subtypes

**Figure 3:**
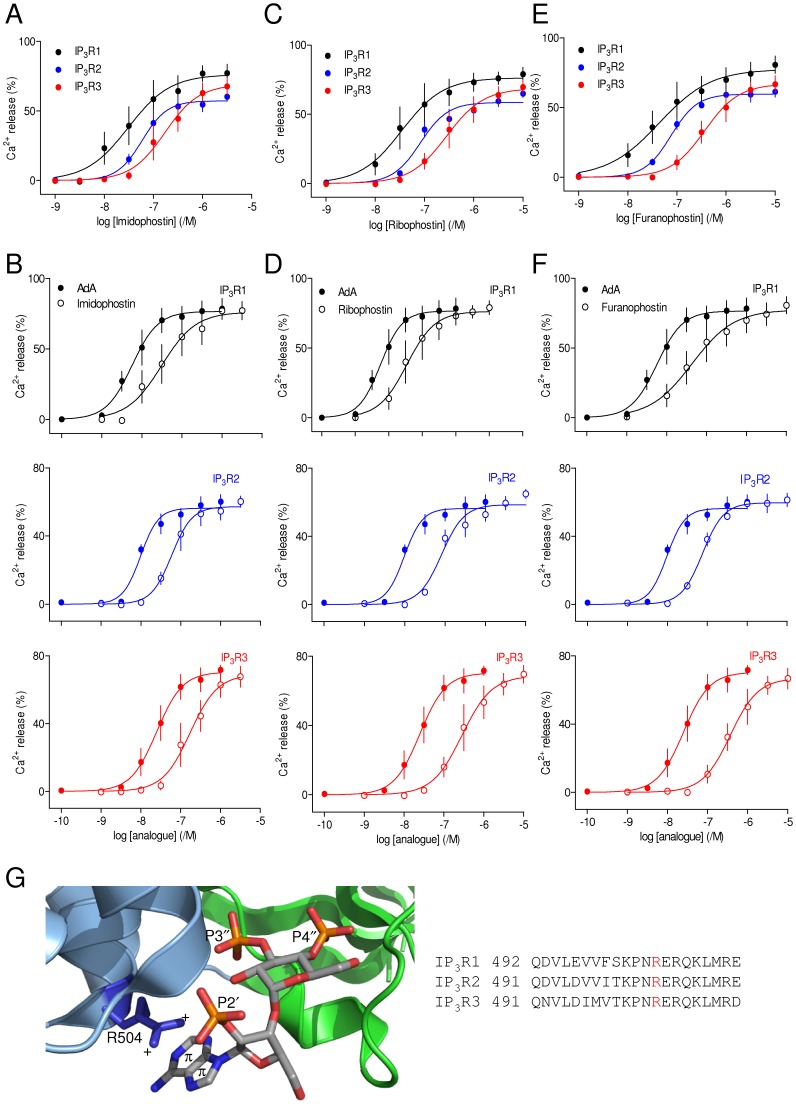
Trimming the adenosine moiety of AdA reduces potency. (A–F) Effects of imidophostin (A), ribophostin (C) and furanophostin (E) on Ca^2+^ release via each of the three IP_3_R subtypes, and the same analogues compared with AdA (B, D and F). Results are means ± S.E.M. from 3 independent experiments. (G) A cation-π interaction between the adenine of AdA and R504 within the α-domain of the IBC is proposed to stabilize AdA binding (left). Closure of the clam-like IBC is proposed to be mediated by interactions between the 3″-phosphate of AdA and the α-domain of the IBC (blue ribbon), and between the 4″-phosphate and the β-domain of the IBC (green ribbon). In 3″-dephospho AdA, a cation-π interaction between AdA and the IBC α-domain is proposed to be sufficient to allow some effective closure of the clam. R504 is conserved in all three mammalian IP_3_R subtypes (right).

Systematic trimming of the adenosine moiety of AdA successively produces imidophostin (which lacks the pyrimidine ring of AdA), ribophostin (in which a methoxy group replaces the adenine moiety of AdA) and furanophostin (in which only the furanoid ring remains) (Figure 1A). Maximally effective concentrations of each of these analogues released the same fraction of the intracellular Ca^2+^ stores as AdA in cells expressing each of the three IP_3_R subtypes, and each analogue was ∼5-10-fold less potent than AdA (Figure 3, Tables 1 and 2). These results are consistent with previous analyses of IP_3_R in hepatocytes, which express predominantly IP_3_R2 [24], [36], with analyses of binding of ribophostin and furanophostin to an N-terminal fragment of IP_3_R1 [12], and with evidence from other analogues that trimming the adenosine moiety decreases affinity for cerebellar IP_3_R, which are largely IP_3_R1 [37].

These results are consistent with our earlier conclusion that the 10-fold greater affinity of AdA relative to IP_3_ requires the adenine moiety of AdA positioned to allow it to form a cation-π interaction with Arg-504 in the α-domain of the IBC of IP_3_R1, a residue that is conserved in all IP_3_R subtypes [8], [12] (Figure 3G). We suggest that this interaction of AdA with IP_3_R is likely to be similar for all IP_3_R subtypes.

### Hydroxyl Moieties that are Important for IP_3_ Binding are Less Important for Binding of AdA

**Figure 4:**
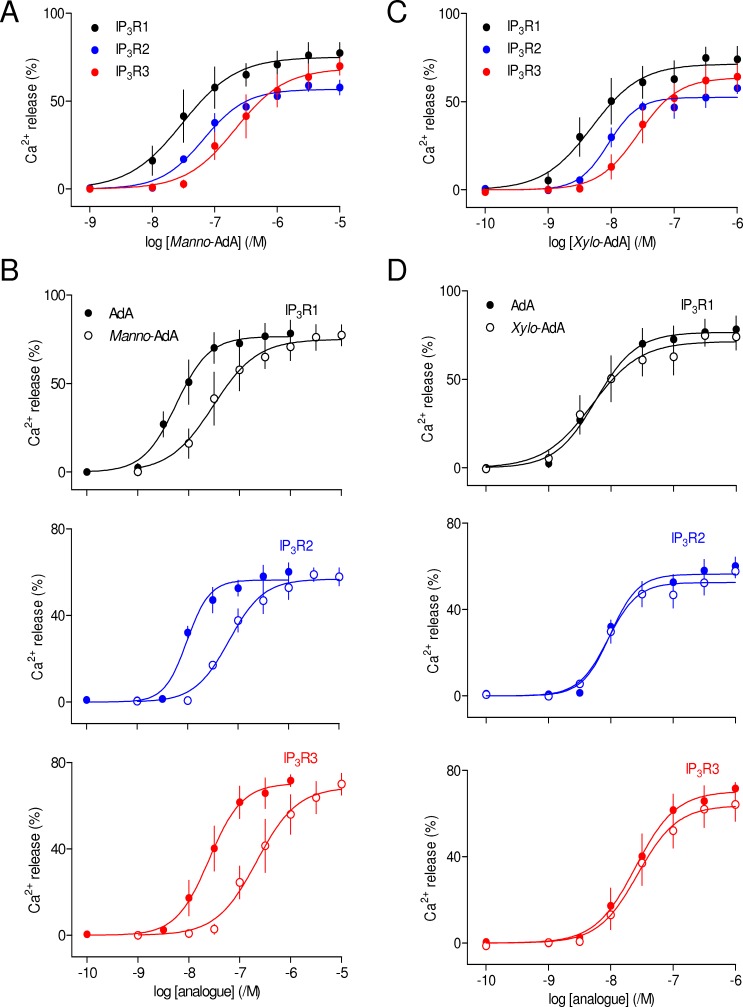
Hydroxyl groups within the glucose ring of AdA are unimportant. (A–D) Effects of *manno*-AdA (A) and *xylo*-AdA (C) on Ca^2+^ release via each IP_3_R subtype, and the same analogues compared with AdA (B and D). Results are means ± S.E.M. from 3 independent experiments.

The 5″-CH_2_OH and 2″-OH substituents of the glucose ring of AdA are thought to mimic the 3-OH and 6-OH of IP_3_, respectively (Figure 1A). A structure equivalent to the 6-OH of IP_3_ is an essential feature of all inositol phosphate analogues that bind to IP_3_R [13], [38], [39] and inversion of its orientation from equatorial to axial reduces affinity by more than 100-fold at all IP_3_R subtypes [40]. It is therefore surprising, but consistent with previous analyses of native hepatic IP_3_R [36], that *manno*-AdA, which differs from AdA only in the orientation of its 2″-OH, should be only 5- to 10-fold less potent than AdA at each IP_3_R subtype (Figures 4A and B, Tables 1 and 2). Why, when the 6-OH of IP_3_ and 2″-OH of AdA seem to be analogous in the ligand structures, should these moieties make such different contributions to the interactions of IP_3_ and AdA with IP_3_R?

The 6-OH of IP_3_ interacts, through a water molecule, with a lysine residue (K569) in the IBC [41] and, by interacting with the adjacent 1-phosphate, it has also been proposed to influence the behaviour of the 4,5-bisphosphate moiety of IP_3_ [42]. The latter interaction is unlikely to contribute to AdA binding because the structures equivalent to the 6-OH (2″-OH of AdA) and the 1-phosphate of IP_3_ (2′-phosphate of AdA) are in different rings in AdA (Figure 1A). We suggest that the lesser importance in AdA of a structure equivalent to the essential 6-OH of IP_3_ comes from this hydroxyl mediating a relatively minor interaction with K569 in AdA, whereas for IP_3_ it contributes also to appropriately orienting the critical 4,5-bisphosphate moiety.

The 3-OH group, although less important than the 6-OH, is another feature of IP_3_ that contributes to high-affinity binding [43]. Our recent analyses of the functional effects of 3-deoxy-IP_3_ established that it was ∼40-fold less potent than IP_3_ at all three IP_3_R subtypes [7]. This is consistent with earlier work showing that 3-deoxy-IP_3_ and analogues with other modifications of the 3-position have reduced affinity for the three IP_3_R subtypes [40]. However, the equivalent modification of AdA, removal of its 5″-CH_2_OH to give *xylo*-AdA (Figure 1A), had no significant effect on its potency at any IP_3_R subtype (Figures 4C and D, Tables 1 and 2). This is consistent with a previous functional analysis of hepatic IP_3_R, where *xylo*-AdA was only marginally less potent than AdA (ΔpEC_50_ ∼0.28) [36]. Our results suggest that despite the apparent structural similarity between the 3-OH of IP_3_ and the 5″-CH_2_OH of AdA (Figure 1A), the two hydroxyl groups do not contribute similarly to ligand binding. Previous analyses of IP_3_ analogues suggested that replacing the 3-OH with the larger CH_2_OH moiety caused the affinity to decrease by no more than 7-fold [40]. A partial explanation for the lack of effect of removing the 5″-CH_2_OH of AdA may therefore be that this moiety is less readily accommodated than a hydroxyl group in the IBC. This would suggest that an analogue of AdA in which the 5″-CH_2_OH is replaced by 5″-OH might bind with increased affinity. We are unaware of such an analogue having been synthesized. The larger substituent at the 5″-position of AdA is, however, unlikely to provide the sole explanation for it making no discernible contribution to binding.

### The 2′-phosphate of AdA is not a Super-optimal Mimic of the 1-phosphate of IP_3_

**Figure 5:**
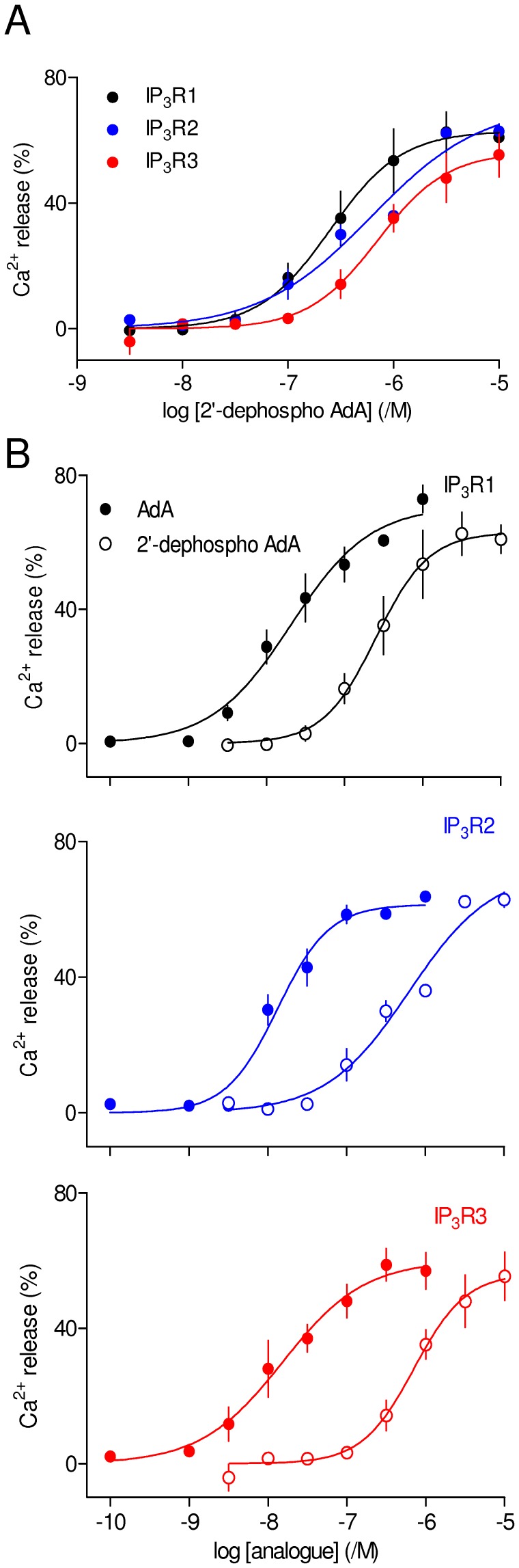
The 2*′*-phosphate of AdA is not the primary cause of its increased potency. (A) Effects of 2*′*-dephospho AdA on Ca^2+^ release via each IP_3_R subtype. (B) The same analogue compared with AdA. Results are means ± S.E.M. from 3–4 independent experiments.

It has been suggested that the 2′-phosphate of AdA interacts with the IBC in a manner that allows it to behave as a super-optimal mimic of the 1-phosphate of IP_3_ [44], [45]. However, our recent study combining structure-activity analyses with mutagenesis of the binding site suggest that the 1-phosphate of IP_3_ is more important for binding than is the 2′-phosphate of AdA [12]. Removal of the 1-phosphate from IP_3_ (to give (4,5)IP_2_) caused its potency and affinity for IP_3_R1 to decrease by ∼100-fold [12], whereas removal of the 2′-phosphate from AdA (2′-dephospho AdA) causes a decrease in potency of ∼17-fold in IP_3_R1 (Figure 5) and ∼40-fold decreases in potency were obtained with 2′-dephospho AdA and IP_3_R2 and IP_3_R3 (Figure 5, Table 1 and 2). These results establish that for all three IP_3_R subtypes, the enhanced affinity of AdA is not due to its 2′-phosphate interacting more effectively than the 1-phosphate of IP_3_ with the IBC.

### A Bisphosphate Moiety is not Essential for Activation of IP_3_ Receptors by AdA

All known active analogues of IP_3_ have structures equivalent to its 4,5-bisphosphate moiety [13]. Structures of the IBC with and without IP_3_ bound provide a rationale for this requirement by revealing that these two phosphate groups contact opposite sides (the α- and β-domains) of the clam-like IBC, closure of which initiates IP_3_R activation [4], [41]. Substantial evidence suggests that the 4″,3″-bisphosphate moiety of AdA mimics the critical 4,5-bisphosphate of IP_3_ [8] (Figure 1A).

**Figure 6:**
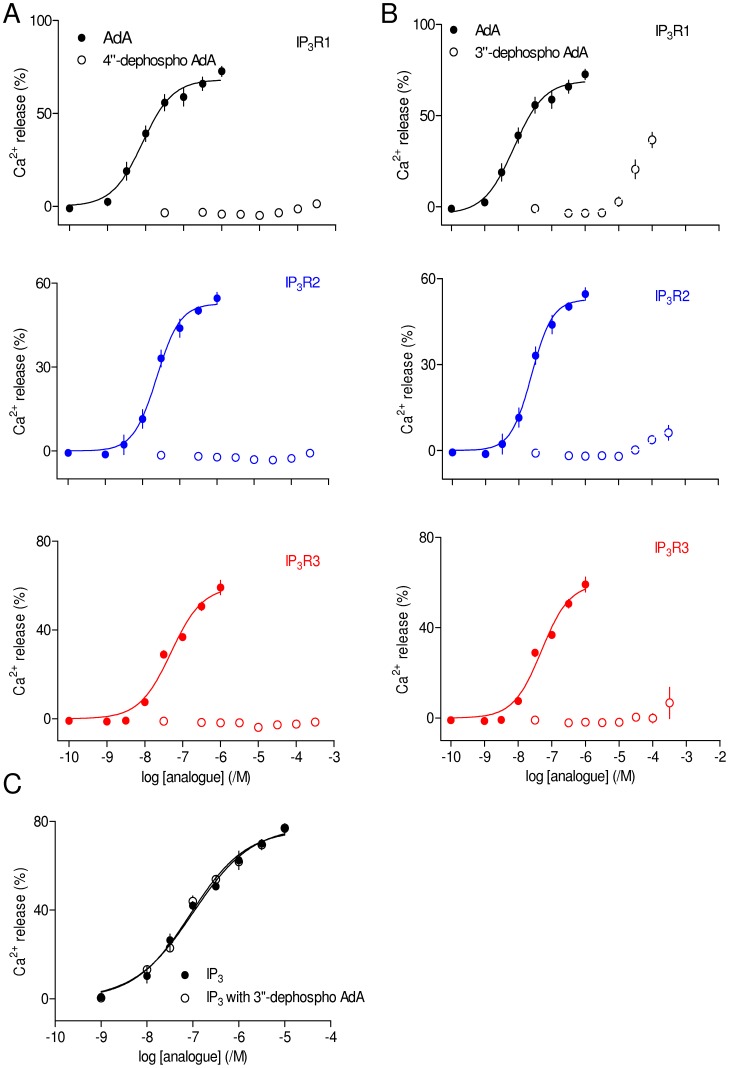
Structures equivalent to the 4,5-bisphosphate of IP_3_ are not essential for AdA activity. (A, B) Concentration-dependent effects on Ca^2+^ release via each IP_3_R subtype of 4″-dephospho AdA (A) and 3″-dephospho AdA (B) compared with AdA. Results are means ± SEM from n independent experiments (n is provided in Table 1). (C) Concentration-dependent effects of IP_3_ alone on Ca^2+^ release via IP_3_R1 or after pre-incubation (30 s) with 3″-dephospho AdA (30 µM), which itself evoked release of 21±5% of the intracellular Ca^2+^ stores. Results (C) are means ± SEM from 3 independent experiments.

4″-dephospho-AdA at concentrations up to 300 µM failed to evoke Ca^2+^ release via any IP_3_R subtype (Figure 6A). This is consistent with previous analyses by both functional and binding assays of IP_3_R1 [28], [46]. 3″-dephospho-AdA did, however, cause detectable Ca^2+^ release albeit with much reduced potency (Figure 6B). The synthetic route used to prepare 3″-dephospho-AdA makes it extremely unlikely that the activity could be due to minor contamination with AdA or related structures with a vicinal bisphosphate moiety. Maximal attainable concentrations of 3″-dephospho-AdA (300 µM) failed to release the entire IP_3_-sensitive Ca^2+^ store, but comparison of the concentrations required to achieve the same submaximal Ca^2+^ release suggests that 3″-dephospho-AdA is ∼10,000-fold less potent than AdA at all three IP_3_R subtypes. With such a massive reduction in potency the lesser sensitivity of DT40-IP_3_R3 cells to AdA means that even the highest practicable concentration of 3″-dephospho-AdA (300 µM) is close to the threshold for detecting Ca^2+^ release (Figure 6B).

The inability of high concentrations of 3″-dephospho-AdA to release the entire IP_3_-sensitive Ca^2+^ store is likely to be due solely to its reduced affinity rather than reduced efficacy. A concentration of 3″-dephospho-AdA (30 µM) that caused detectable Ca^2+^ release via IP_3_R1 (∼21±5%) had no effect on the sensitivity of the Ca^2+^ release evoked by a subsequent addition of IP_3_. The pEC_50_ was 7.00±0.02 and 7.04±0.06 (n = 3) for (1,4,5)IP_3_ alone and in the presence of 3″-dephospho-AdA, respectively (Figure 6C). A partial agonist would be expected to shift the sensitivity to higher concentrations of IP_3_. These results suggest that 3″-dephospho-AdA is a low-affinity full agonist of IP_3_R.

These results extend our previous analyses of IP_3_R1 by demonstrating that for all IP_3_R subtypes, the 4″-phosphate group of AdA is essential for activity, whereas the 3″-phosphate is important but not essential. 3″-dephospho-AdA is the only known agonist of IP_3_R to lack a structure equivalent to the 4,5-bisphosphate moiety of IP_3_.

## Discussion

AdA is a high-affinity full agonist of IP_3_R that has been extensively used to explore the behaviour of IP_3_R [reviewed in8]. The activity of AdA has been confirmed in many cell types, but hitherto there has been no assessment of its activity in homogenous populations of IP_3_R subtypes. We have demonstrated that AdA is ∼10-fold more potent than IP_3_R at each IP_3_R subtype (Figure 2, Tables 1 and 2), and the structural determinants of its high-affinity interaction with IP_3_R are similar for all three IP_3_R subtypes. Contrary to an earlier suggestion that the 2′-phosphate of AdA mediates its enhanced affinity by forming a stronger interaction with the IBC than the analogous 1-phosphate of IP_3_, we find that the 1-phosphate makes a greater contribution to IP_3_ binding than does the 2′-phosphate of AdA (Figure 5) [12]. A more likely explanation for the enhanced affinity of AdA is a cation-π interaction between its adenine moiety and R504 within the α-subunit of the IBC (Figure 3G) [28]. That explanation is supported by results for each IP_3_R subtype showing that truncation of the adenosine moiety of AdA brings the potency of the resulting analogues (imidophostin, ribophostin and furanophostin) close to that of IP_3_ (Figure 3).

A key step in the initial activation of IP_3_R by IP_3_ appears to be closure of its clam-like IBC as the 4-phosphate of IP_3_ contacts one side of the clam (its β-domain) and the 5-phosphate contacts the other side (α-domain) [4]. That mechanism provides a satisfying explanation for the long-standing observation that all inositol phosphates that activate IP_3_R share this essential 4,5-bisphosphate moiety. AdA is different in that its 4″-phosphate (analogous to the 4-phosphate of IP_3_, Figure 1A) is essential, but 3″-dephospho-AdA retains activity at all three IP_3_R subtypes, albeit with very low affinity (Figure 6). We suggest that for AdA, the need for the bisphosphate moiety to cause closure of the IBC can be partially replaced for all IP_3_R subtypes by having an interaction between the adenine of AdA and the α-domain substitute for the interaction between the 3″-phosphate (analogous to the 5-phosphate of IP_3_) and the α-domain [28]. Finally, whereas the 6-OH and, to a lesser extent, the 3-OH of IP_3_ are important for IP_3_ binding, the equivalent structures within AdA play lesser roles.

Both store-operated Ca^2+^ entry, which is triggered by depletion of IP_3_-sensitive Ca^2+^ stores [47], and the spatial organization of subcellular Ca^2+^ signals have been reported to be differentially affected by IP_3_, AdA or its analogues [14], [16], [17], [19], [21], [22]. Our present results, which demonstrate that AdA structure-activity relationships are similar for all IP_3_R subtypes, suggest that different physiological effects of IP_3_, AdA or its analogues are more likely to result from differences in their affinities, kinetics or rates of degradation than from selective interactions with different IP_3_R subtypes.
